# Marker-assisted breeding of the rice restorer line Wanhui 6725 for disease resistance, submergence tolerance and aromatic fragrance

**DOI:** 10.1186/s12284-016-0139-9

**Published:** 2016-12-01

**Authors:** Yanchang Luo, Tingchen Ma, Aifang Zhang, Kar Hui Ong, Zefu Li, Jianbo Yang, Zhongchao Yin

**Affiliations:** 1 Temasek Life Sciences Laboratory, 1 Research Link, National University of Singapore, Singapore, 117604 Republic of Singapore; 2 Key Laboratory of Rice Genetics and Breeding, Anhui Rice Research Institute, Anhui Academy of Agricultural Sciences, Hefei, 230031 China; 3 Plant Protection Research Institute, Anhui Academy of Agricultural Sciences, Hefei , 230031, , China; 4 Department of Biological Sciences, 14 Science Drive, National University of Singapore, Singapore, 117543 Republic of Singapore

**Keywords:** *badh2.1*, *Pi9*, *Sub1A*, *Xa4*, *Xa21*, *Xa27*, Mianhui 725, Wanhui 6725, Marker-assisted selection

## Abstract

**Background:**

Rice is a staple food crop in the world. With the increase in world population and economic development, farmers need to produce more rice in limited field. However, the rice production is frequently affected by biotic and abiotic stresses. The use of natural disease resistance and stress tolerance through genetic breeding is the most efficient and economical way to combat or acclimate to these stresses. In addition, rice with aromatic fragrance can significantly increase market value for its good grain quality. Mianhui 725 (MH725) is an elite restorer line that has been widely used to produce three-line hybrid rice in China. We previously introduced rice bacterial blight resistance genes *Xa4* and *Xa21* into MH725 and obtained an introgression rice line Wanhui 421 (WH421), which theoretically possesses 96.9% genetic background of MH725.

**Results:**

Here we report the introduction and pyramiding of disease resistance genes *Xa27* and *Pi9*, submergence tolerance gene *Sub1A* and aromatic fragrance gene *badh2.1* in WH421 through backcrossing and marker-assisted selection. The newly developed introgression rice line was designated as Wanhui 6725 (WH6725), which theoretically possesses 95.0% genetic background of MH725. WH6725 and its hybrid rice conferred disease resistance to both blast and bacterial blight diseases and showed tolerance to submergence for over 14 days without significant loss of viability. Compared with non-aromatic rice MH725, WH6725 has strong aromatic fragrance. The major important agronomic traits and grain quality of WH6725 and its hybrid rice obtained in field trials were similar to those of MH725 and the control hybrid rice, indicating that WH6725 is as good as MH725 when it is used as a restorer line for three-line hybrid rice production.

**Conclusion:**

We have successfully developed a new restorer line WH6725 with disease resistance to rice blast and bacterial blight, tolerance to submergence and aromatic fragrance, which can be used to replace MH725 for hybrid rice production.

## Background

Rice (*Oryza sativa* L.) is a staple food crop feeding more than three billion people. Rice yield had gone through two big leaps within a period of four decades from the 1960s to 1990s (Zhang 2007). The first leap was the use of semi-dwarf gene, which reduced plant height and thereby increased the harvest index (Spielmeyer et al. 2002). The second leap was the development of hybrid rice (Yuan et al. 1994). The rapid population growth and economic development have been posing a growing pressure on rice researchers and farmers for increasing rice production in limited rice field. However, the rice production is frequently affected by biotic stress, including various diseases caused by pests and pathogens, and abiotic stress, such as drought and flood due to the global warming and climate change.

Bacterial blight, caused by *Xanthomonas oryzae* pv. *oryzae*, is one of the devastating bacterial diseases in rice production (Gnanamanickam et al. 1999). The disease could result in 50% yield reduction in severe cases if plants are infected by *X. oryzae* pv. *oryzae* strains at the maximum tillering stage (Mew et al. 1993). Rice blast, caused by the fungus *Magnaporthe oryzae*, is the most important fungal disease in rice production and its repercussion is the yield loss of 157 million tons of rice annually in the world (Kaundal et al. 2006). During the evolution, rice has co-evolved disease resistance (*R*) genes against the infection by the two kinds of pathogens (Liu et al. 2014). The utilization of host disease resistance (*R*) genes is still an efficient and economic method for controlling diseases. *Xa4*, *Xa21* and *Xa27* are three dominant *R* genes in rice that provide race-specific and broad-spectrum resistance to *X. oryzae* pv. *oryzae* (Gu et al. 2004; Ikeda et al. 1990; Sun et al. 2003). Cultivars with the *Xa4* gene were resistant to almost all Chinese pathotypes of *X. oryzae* pv. *oryzae* except for pathotype C5 (Zhang 2009). The *Xa4* gene has been widely used in breeding of parental lines of hybrid rice in China since 1980s (Zhang 2009). Cultivar IRBB21, carrying *Xa21* in IR24 genetic background, conferred resistance to all the known *X. oryzae* pv. *oryzae* strains collected from India and Philippines (Ikeda et al. 1990). Since the late 1990s, the *Xa21* gene has been widely incorporated in Asian rice for bacterial blight resistance (Datta et al. 2002; Huang et al. 1997; Kottapalli et al. 2010; Luo and Yin 2013; Luo et al. 2014; Perez et al. 2008; Rajpurohit et al. 2011; Singh et al. 2001; Zhai et al. 2002; Zhang et al. 2006). IRBB27, which carries *Xa27* in IR24 genetic background, was resistant or moderately resistant to 30 of 35 *X. oryzae* pv. *oryzae* strains collected from 11 countries (Gu et al. 2004). The *Xa27* gene had been introduced into the paternal line of hybrid rice (Luo et al. 2012). The rice blast *R* gene *Pi9* in the indica rice line 75-1-127 was introgressed from the wild species *Oryza minuta* (Amante-Bordeos et al. 1992; Liu et al. 2002)*.* The *Pi9* gene conferred broad-spectrum disease resistance to 43 *M. oryzae* isolates collected from 13 countries (Liu et al. 2002) and was used in rice breeding program (Khanna et al. 2015; Koide et al. 2011; Luo and Yin 2013; Ni et al. 2015).

Drought and flood are the two major abiotic stresses for rice production, especially in rainfed lowland ecosystem, and both stresses can occur alternatively during a single crop cycle (Fukao et al. 2011). Rice variety FR13A is tolerant to submergence (Xu and Mackill 1996). A major locus *Sub1A* for submergence tolerance was mapped onto rice chromosome 9 (Xu and Mackill 1996) and cloned (Xu et al. 2006). *Sub1A* encodes an ethylene responsive transcription factor (SUB1A) whose function is to dampen ethylene production and gibberellic acid responsiveness during submergence, so as to economically use carbohydrate reserves and to prolong rice plants to submergence (Xu et al. 2006). Interestingly, SUB1A also enhances the recovery of plants from drought at the vegetative stage through the decrease of leaf water evaporation, lipid peroxidation and the increase of gene expression related with acclimation to dehydration (Fukao et al. 2011). Over-expression of *Sub1A* enhances abscisic acid responsiveness, thereby activating stress-inducible gene expression (Fukao et al. 2011). The *Sub1A* gene was deployed in broadly grown Asian rice cultivar Swarna and Thai fragrance rice Khao Dawk Mali 105 (KDML105) (Luo and Yin 2013; Neeraja et al. 2007).

The fragrance of aromatic rice is an important agronomic trait that determines the premium price in global rice market. KDML 105, commonly known as Thai Hom Mali rice or *jasmine* rice, is the most popular aromatic rice cultivar mainly grown in Thailand (Sarkarung et al. 2000; Somrith 1996). An 8-bp deletion in the exon 7 of the *Badh2* gene (*badh2.1*) resulted in the truncation of BADH2 enzyme in KDML105 (Bradbury et al. 2005). The loss-of-function of BADH2 enzyme leads to the accumulation of an aromatic compound, 2-acetyl-1-pyrroline, in fragrant rice (Bradbury et al. 2005; Kovach et al. 2009). Two PCR-based DNA markers M265 and M355 were developed to detect the *badh2.1* allele from KDML105 and the *Badh2* alleles from non-aromatic rice cultivars, respectively (Luo and Yin 2013).

Marker-assisted selection (MAS) is an indirect selection process in molecular breeding. A trait of interest is selected based on DNA-based molecular markers co-segregated with or derived from portions of the gene that controls the trait rather than the trait itself. MAS is a precise and efficient selection system that allows for recessive allele selection, early stage selection and multiple genes pyramiding without traditional phenotypic evaluation for each trait. The development of molecular markers for the selection of genes is a goal for many rice breeding programs (Blair and McCouch 1997). The gene sequences and the whole rice genome sequence provide a powerful platform for developing simple and precise molecular markers for MAS.

Mianhui 725 (MH725) is an elite restorer line with good grain quality that has been widely used to produce three-line hybrid rice in China (Luo et al. 2012). However, MH725 is susceptible to many *X. oryzae* pv. *oryzae* strains (Luo et al. 2012) and *M. oryzae* isolates from China and the Philipinnes (Wang and He 2007). We previously introduced the *Xa4* and *Xa21* genes into MH725 and obtained an introgression rice line Wanhui 421 (WH421), which theoretically possesses 96.9% genetic background of MH725 (Luo et al. 2012). Here we report further introduction and pyramiding of the *badh2.1*, *Pi9*, *Sub1A*, and *Xa27* genes into WH421 through recurrent backcrossing, gene pyramiding and maker-assisted selection. Our objective was to develop a new restorer rice line mainly in MH725 genetic background for hybrid rice production with disease resistance to rice blast and bacterial blight, submergence tolerance and aromatic fragrance.

## Results

### Breeding of WH6725

**Fig. 1:**
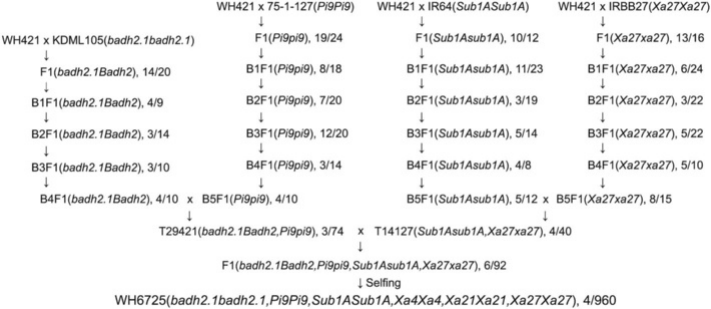
Strategy for breeding of WH6725. WH421 (genotype: *Xa4Xa4,Xa21Xa21*) was used as the recurrent female line for crossing and backcrossing. The number of positive plants over the number of total plants screened are indicated in each generation. For gene pyramiding, four F2 plants containing homozygous alleles at all loci were obtained in the F2 population and one of the plants, F2-281, was designated as Wanhui 6725 (WH6725). The genotypes of *Xa4* and *Xa21* genes are only shown in WH6725 but not in other generations

**Fig. 2:**
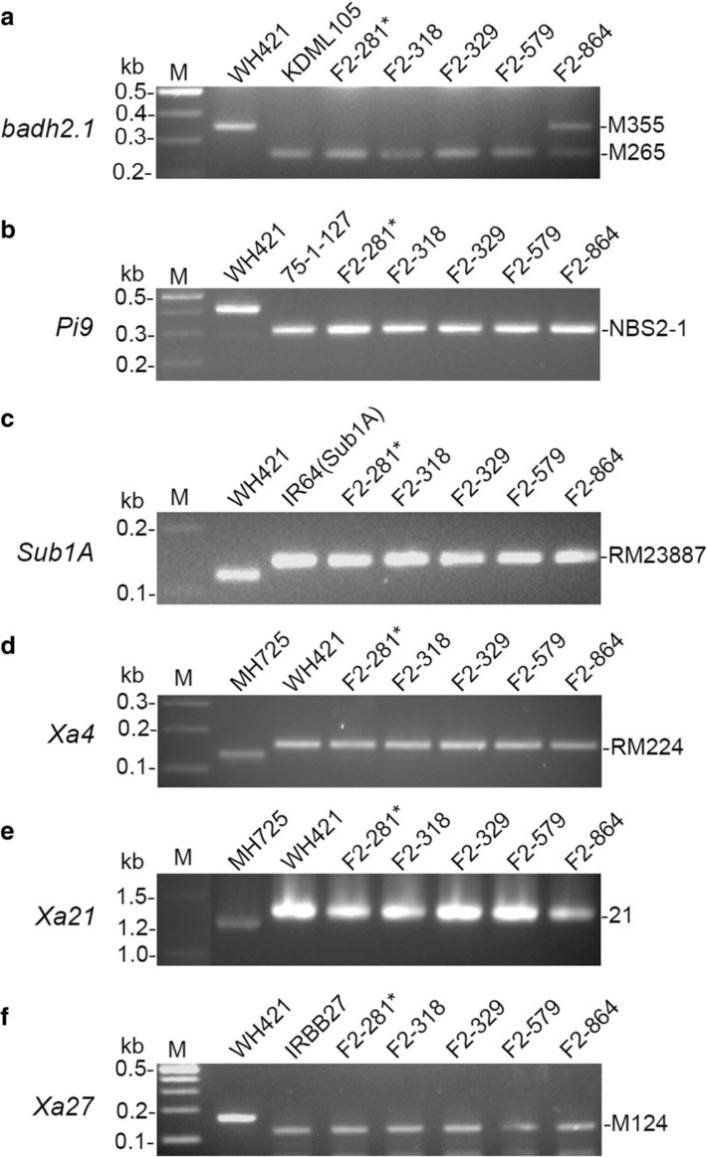
Detection of patterns of molecular markers in rice lines. **a** Patterns of allele-specific markers M265 (for the *badh2.1* allele) and M355 (for the *Badh2* allele) at the *Badh2* locus. **b** Patterns of co-dominant marker NBS2-1 at the *Pi9* locus. **c** Patterns of co-dominant marker RM23887 linked with the *Sub1A* locus. **d** Patterns of co-dominant marker RM224 linked with the *Xa4* locus. **e** Patterns of co-dominant marker 21 at the *Xa21* locus. **f** Patterns of co-dominant marker M124 co-segregated the *Xa27* locus. Plant F2-281, marked with an asterisk, was selected and designated as Wanhui 6725 (WH6725) for further study

A two-step breeding approach was employed to introduce and pyramid the *badh2.1*, *Pi9*, *Sub1A* and *Xa27* genes in WH421 genetic background (Fig. 1). The first step was to cross WH421 (female) with KDML105 (*badh2.1badh2.1*), 75-1-127(*Pi9Pi9*), IR64(*Sub1ASub1A*) or IRBB27(*Xa27Xa27*) followed by 4–5 rounds of backcrossing using WH421 as the recurrent female line (Fig. 1). The second step was to pyramid the 6 genes, including the *Xa4* and *Xa21* genes from WH421, in a single introgression line mainly in MH725 genetic background (Fig. 1). At each generation, the genotypes at the *badh2.1*, *Pi9*, *Sub1A* and *Xa27* loci in the cross and backcross progeny were determined by molecular markers M265/M355, NBS2-1, RM23887 and M124, respectively (Table 1). About 3–19 positive plants were obtained from 8 to 24 progeny of different crosses or backcrosses (Fig.1). The genotypes of the *Xa4* and *Xa21* loci in the backcross progeny were determined by molecular markers RM224 and 21, respectively (Table 1). The backcross progeny that contained homozygous *Xa4* and *Xa21* genes and the target gene (*badh2.1*, *Pi9*, *Sub1A* or *Xa27*) were selected for further backcrossing (data not shown). Theoretically, after 4–5 rounds of backcrossing, the BC4F1 and BC5F1 plants would have possessed 96.9 and 98.4% of the genetic background from the recurrent female parent WH421, respectively. In addition, the morphological phenotypes and growth duration of plants B4F1(*badh2.1BadH2*), B5F1(*Pi9pi9*), B5F1(*Sub1Asub1A*) and B5F1 (*Xa27xa27*) were similar to that of WH421 (data not shown). To combine the *badh2.1* and *Pi9* genes in a single plant, a B4F1(*badh2.1Badh2*) plant was crossed with a WH421 B5F1(*Pi9pi9*) plant to produce plant T29421(*badh2.1Badh2*,*Pi9pi9*) (Fig. 1). Similarly, a B5F1(*Sub1Asub1A*) plant was crossed with a B5F1(*Xa27xa27*) plant to produce plant T14127(*Sub1Asub1A, Xa27xa27*) (Fig. 1). The plant T29421(*badh2.1Badh2*,*Pi9pi9*) was then crossed with the plant T14127(*Sub1Asub1A, Xa27xa27*). Ninety-two F1 plants were produced from the cross. After genotyping, 6 of them were identified to carry heterozygous alleles at the *badh2.1*, *Pi9*, *Sub1A* and *Xa27* loci (Fig. 1). The 6 F1 plants were self-crossed to generate F2 populations that collectively contained 960 individuals. After genotyping, 4 F2 plants, F2–281, F2–318, F2–329 and F2–579, were found to carry homozygous alleles at the *badh2.1*, *Pi9*, *Sub1A*, *Xa4*, *Xa21* and *Xa27* loci (Fig. 2). The 4 F2 plants showed similar morphological phenotype in growth and development when they were grown in greenhouse (data not shown) and one of them, F2–281, was designated as Wahhui 6725 (WH6725) (genotype: *badh2.1badh2.1,Pi9Pi9*,*Sub1ASub1A*,*Xa4Xa4*,*Xa21Xa21*,*Xa27Xa27*). Theoretically, WH6725 possesses 95.0% genetic background of MH725. Therefore, we selected MH725 and its hybrid rice as the controls for WH6725 and its hybrid rice in the subsequent experiments.

**Table 1:** Molecular markers used in this study

Marker	Gene of interest	DNA sequence (5’ to 3’)^a^	Type of marker^b^	Reference
M265	*badh2.1*	F: ACCAGGACTTGTTTGGAGCTTG	STS, dominant	(Luo and Yin 2013)
		R: CCATAGGAGCAGCTGAAATATATACC		
M355	*Badh2*	F: CTGGTAAAAAGATTATGGCTTCA	STS, dominant	(Luo and Yin 2013)
		R: AGTGCTTTACAAAGTCCCGCAC-3’		
NBS2-1	*Pi9*	F: GGATTCGACAGATGGTGCAACAAC	STS, co-dominant	This study
		R: ACATCCACCATCCAAACGGGAAAC		
RM23887	*Sub1A*	F: TCGACCCAATATCTTTCTGC	SSR, co-dominant	(Neeraja et al. 2007)
		R: CTGTCTGTTCACTTGTGTTCACC		
RM224	*Xa4*	F: ATCGATCGATCTTCACGAGG	SSR, co-dominant	(Sun et al. 2003)
		R: TGCTATAAAAGGCATTCGGG		
21	*Xa21*	F: ATAGCAACTGATTGCTTGG	STS, co-dominant	(Chen et al. 2000)
		R: GATCGGTATAACAGCAAAAC		
M124	*Xa27*	F: ATCTGGAGCAGAGCTTAAGGTGTG	STS, co-dominant	This study
		R: AGCAGTTCTCATATAAATGTTGGTTG		

^a^ *F* forward primer; *R*, reverse primer.

^b^ *STS* sequence-tagged site; *SSR*, simple sequence repeat

### Disease resistance of WH6725 and its hybrid rice to rice blast and bacterial blight

**Fig. 3:**
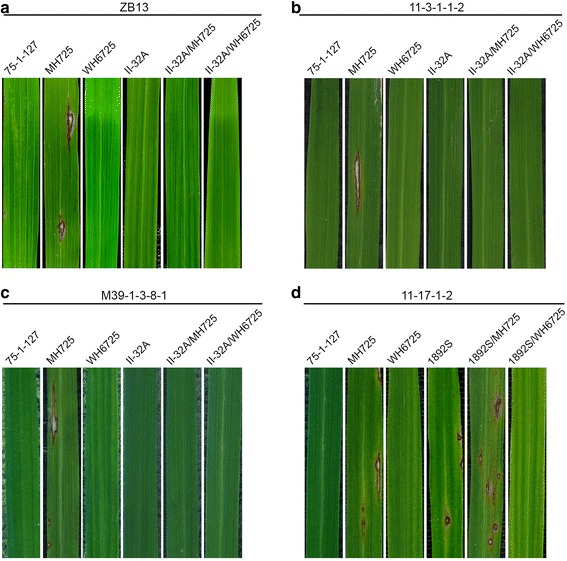
Evaluation of rice lines for disease resistance to rice blast. The 4-leaf-stage rice seedlings were inoculated with *M. oryzae* isolates ZB13 **a**,11-3-1-1-2 **b**, M39-1-3-8-1 **c** and 11-17-1-2 **d**. The images of inoculated leaves were taken at 7 days after inoculation

The rice lines, including 75-1-127, MH725, WH6725, the cytoplasmic male sterile (CMS) line II-32A and the thermosensitive-genic male sterility (TGMS) line 18892S, and their hybrid rice (II-32A/MH725, II32A/WH6725, 1892S/MH725 and 1892S/WH6725) were evaluated for rice blast resistance with *M. oryzae* isolates collected from China (ZB13, 11-3-1-1-2 and M39-1-3-8-1) or the Philippines (11-17-1-2). MH725 was susceptible to ZB13, 11-3-1-1-2 and M39-1-3-8-1, whereas 75-1-127 and WH6725 were resistant to the 3 *M. oryzae* isolates (Fig. 3a, b, c). Since the CMS line II-32A is resistant to ZB13, 11-3-1-1-2 and M39-1-3-8-1, we were not able to test the function of the *Pi9* gene in 3-line hybrid rice as both II-32A/MH725 and II-32A/WH6725 showed resistance to the 3 *M. oryzae* isolates (Fig. 3a, b and c). Thus, in order to evaluate the function of the *Pi9* gene in hybrid rice background, we made two two-line hybrids, 1892S/MH725 and 1892S/WH6725, by crossing the TGMS line 1892S with MH725 and WH6725, respectively, and tested their resistance to the *M. oryzae* isolate 11-17-1-2. MH725, 1892S and 1892S/MH725 were susceptible to 11-17-1-2, whereas the *Pi9* donor line 75-1-127 was resistant to the *M. oryzae* isolate (Fig. 3d). To our expectation, both WH6725 and 1892S/WH6725 were resistant to 11-17-1-2 (Fig. 3d). The results demonstrated that the *Pi9* gene in the F1 hybrids, which only harboured heterozygous *Pi9* gene derived from WH6725, could provide full disease resistance to rice blast.

Evaluation of MH725, WH421, IRBB27, WH6725, II-32A, II-32A/MH725 and II-32A/WH6725 for disease resistance to bacterial blight was carried out using 27 *X. oryzae* pv. *oryzae* strains collected from 10 countries (Table 2). MH725 was susceptible or moderately susceptible to 26 *X. oryzae* pv. *oryzae* strains and was only moderately resistant to HLJ72 (Table 2). WH421 was resistant or moderately resistant to 22 *X. oryzae* pv. *oryzae* strains but was still susceptible to A3842, JW89011, NXO260, PXO99(R6) and Thai R-7 (Table 2). IRBB27 conferred high resistance to 24 *X. oryzae* pv*. oryzae* strains but was susceptible to ZHE173, K202 and Thai R-2 (Table 2). WH6725 conferred high resistance to all 27 *X. oryzae* pv*. oryzae* strains tested (Table 2). The CMS line II-32A was susceptible to 24 *X. oryzae* pv*. oryzae* strains but was still resistant or moderately resistant to Aust-R3, LN57 and PXO112(R5) (Table 2). The control F1 hybrid II-32A/MH725 was susceptible to 23 *X. oryzae* pv*. oryzae* strains but was still resistant or moderately resistant to Aust-R3, HLJ72, LN57 and PXO112(R5) (Table 2). Similar to the improved paternal line WH6725, the F1 hybrid II-32A/WH6725 was highly resistant to all 27 *X. oryzae* pv. *oryzae* strains tested (Table 2). The results demonstrated that the *Xa4*, *Xa21* and *Xa27* genes in WH6725 and its F1 hybrids provided high and broad-spectrum disease resistance to multiple *X. oryzae* pv. *oryzae* strains.

**Table 2:** Disease evaluation of rice lines for resistance to rice bacterial blight

Strain	Origin	Lesion length (cm) and resistance score^a^						
		MH725	WH421	IRBB27	WH6725	II-32A	II-32A/MH725	II-32A/WH6725
Aust-2031	Australia	34.0 ± 3.4 (S)	4.5 ± 1.4 (MR)	0.4 ± 0.3 (R)	0.2 ± 0.1 (R)	12.0 ± 8.4 (S)	12.5 ± 2.1 (S)	0.1 ± 0.0 (R)
Aust-R3	Australia	21.4 ± 3.2 (S)	4.3 ± 1.5 (MR)	0.1 ± 0.0 (R)	0.2 ± 0.0 (R)	0.2 ± 0.1 (R)	0.2 ± 0.1 (R)	0.1 ± 0.0 (R)
GD1358	China	9.5 ± 0.7 (S)	2.6 ± 1.9 (R)	0.2 ± 0.1 (R)	0.2 ± 0.1 (R)	16.3 ± 10.6 (S)	15.1 ± 1.9 (S)	0.2 ± 0.1 (R)
HB17	China	9.6 ± 1.6 (S)	3.2 ± 2.3 (MR)	0.9 ± 1.2 (R)	0.1 ± 0.1 (R)	26.8 ± 7.7 (S)	33.6 ± 4.7 (S)	1.3 ± 0.5 (R)
HB21	China	14.2 ± 5.3 (S)	3.3 ± 1.5 (MR)	0.2 ± 0.1 (R)	0.1 ± 0.1 (R)	25.3 ± 3.2 (S)	10.5 ± 0.4 (S)	0.1 ± 0.1 (R)
HLJ72	China	4.4 ± 0.7 (MR)	2.8 ± 1.6 (R)	2.9 ± 1.2 (R)	0.1 ± 0.1 (R)	16.3 ± 3.0 (S)	0.4 ± 0.3 (R)	0.1 ± 0.0 (R)
JS49-6	China	41.7 ± 5.1 (S)	2.9 ± 1.7 (R)	0.1 ± 0.1 (R)	0.2 ± 0.0 (R)	22.6 ± 2.8 (S)	24.2 ± 5.3 (S)	0.2 ± 0.1 (R)
LN57	China	8.5 ± 4.4 (MS)	3.3 ± 1.4 (MR)	0.3 ± 0.2 (R)	0.2 ± 0.1 (R)	1.1 ± 0.8 (R)	0.2 ± 0.1 (R)	0.1 ± 0.0 (R)
NX42	China	17.9 ± 4.5 (S)	3.7 ± 2.0 (MR)	0.3 ± 0.6 (R)	0.1 ± 0.1 (R)	25.4 ± 5.1 (S)	27.6 ± 2.5 (S)	2.2 ± 0.6 (R)
ZHE173	China	27.9 ± 5.4 (S)	3.8 ± 4.2 (MR)	18.4 ± 2.6 (S)	0.2 ± 0.1 (R)	21.4 ± 3.0 (S)	23.9 ± 3.6 (S)	2.5 ± 0.7 (R)
CIAT1185	Columbia	13.7 ± 1.5 (S)	4.7 ± 3.7 (MR)	0.2 ± 0.1 (R)	0.1 ± 0.1 (R)	20.1 ± 2.9 (S)	14.7 ± 3.1 (S)	0.1 ± 0.1 (R)
A3842	India	30.7 ± 4.3 (S)	18.0 ± 7.9 (S)	0.2 ± 0.1 (R)	1.1 ± 1.3 (R)	25.4 ± 3.6 (S)	35.0 ± 3.2 (S)	0.2 ± 0.1 (R)
A3857	India	9.6 ± 1.0 (S)	5.8 ± 1.8 (MR)	0.1 ± 0.0 (R)	0.2 ± 0.1 (R)	27.3 ± 4.5 (S)	27.7 ± 7.5 (S)	0.2 ± 0.1 (R)
IXO56	Indonesia	18.6 ± 5.6 (S)	5.9 ± 3.7 (MR)	0.2 ± 0.1 (R)	0.2 ± 0.1 (R)	27.4 ± 3.0 (S)	30.9 ± 3.8 (S)	2.2 ± 1.2 (R)
H75373	Japan	9.7 ± 3.0 (S)	5.3 ± 4.9 (MR)	1.0 ± 1.2 (R)	0.2 ± 0.1 (R)	26.6 ± 3.5 (S)	29.0 ± 3.7 (S)	0.2 ± 0.1 (R)
T7174	Japan	18.6 ± 3.2 (S)	2.7 ± 2.0 (R)	0.1 ± 0.0 (R)	0.2 ± 0.1 (R)	15.0 ± 5.0 (S)	10.2 ± 0.5 (S)	0.1 ± 0.0 (R)
JW89011	Korea	14.7 ± 3.2 (S)	10.7 ± 9.6 (S)	0.5 ± 0.6 (R)	1.1 ± 1.2 (R)	23.1 ± 4.6 (S)	11.3 ± 0.6 (S)	0.1 ± 0.0 (R)
K202	Korea	17.0 ± 4.5 (S)	2.7 ± 1.1 (R)	18.7 ± 5.5 (S)	0.3 ± 0.2 (R)	30.7 ± 2.8 (S)	35.0 ± 3.4 (S)	2.5 ± 0.8 (R)
NXO260	Nepal	12.7 ± 0.7 (S)	9.2 ± 3.6 (S)	0.3 ± 0.2 (R)	0.1 ± 0.1 (R)	30.1 ± 2.0 (S)	29.5 ± 2.5 (S)	0.1 ± 0.1 (R)
PXO86(R2)	Philippines	8.0 ± 4.9 (MS)	1.3 ± 1.0 (R)	0.3 ± 0.2 (R)	0.2 ± 0.1 (R)	22.6 ± 2.7 (S)	17.9 ± 4.1 (S)	0.2 ± 0.1 (R)
PXO79(R3)	Philippines	12.9 ± 4.2 (S)	1.5 ± 1.1 (R)	0.3 ± 0.1 (R)	0.2 ± 0.1 (R)	17.4 ± 2.1 (S)	15.3 ± 2.5 (S)	0.2 ± 0.1 (R)
PXO71(R4)	Philippines	9.4 ± 3.1 (S)	2.2 ± 1.4 (R)	0.6 ± 0.3 (R)	0.2 ± 0.1 (R)	26.9 ± 2.7 (S)	24.4 ± 8.8 (S)	0.1 ± 0.1 (R)
PXO113(R4)	Philippines	30.1 ± 5.6 (S)	0.1 ± 0.1 (R)	0.2 ± 0.2 (R)	0.1 ± 0.1 (R)	25.8 ± 3.6 (S)	11.4 ± 9.1 (S)	0.1 ± 0.1 (R)
PXO112(R5)	Philippines	25.4 ± 4.7 (S)	0.8 ± 0.7 (R)	0.1 ± 0.1 (R)	0.1 ± 0.1 (R)	4.0 ± 1.0 (MR)	0.3 ± 0.1 (R)	0.1 ± 0.1 (R)
PXO99(R6)	Philippines	19.9 ± 5.5 (S)	16.3 ± 5.9 (S)	0.1 ± 0.0 (R)	0.1 ± 0.0 (R)	27.9 ± 2.8 (S)	25.3 ± 4.8 (S)	1.5 ± 1.5 (R)
Thai R-7	Thailand	34.0 ± 3.4 (S)	14.5 ± 6.0 (S)	0.5 ± 0.8 (R)	0.1 ± 0.1 (R)	20.3 ± 3.8 (S)	18.4 ± 4.9 (S)	0.8 ± 0.9 (R)
Thai R-2	Thailand	21.4 ± 3.2 (S)	4.4 ± 2.3 (MR)	22.6 ± 3.5 (S)	0.1 ± 0.1 (R)	18.6 ± 5.6 (S)	22.4 ± 2.2 (S)	2.4 ± 0.4 (R)

^a^The lesion length (L.L.) of bacterial blight is the average of 16 infected leaves from 4 inoculated plants. The standard deviation of the mean is indicated. For disease score: R, resistant, lesion length (L.L.) ≤ 3.0 cm; MR, moderately resistant, 3.0 cm < L.L. ≤ 6.0 cm; MS, moderately susceptible, 6.0 cm < L.L. ≤ 9.0 cm; S, susceptible, L.L. > 9.0 cm

### Submergence tolerance of WH6725 and its hybrid rice

**Fig. 4:**
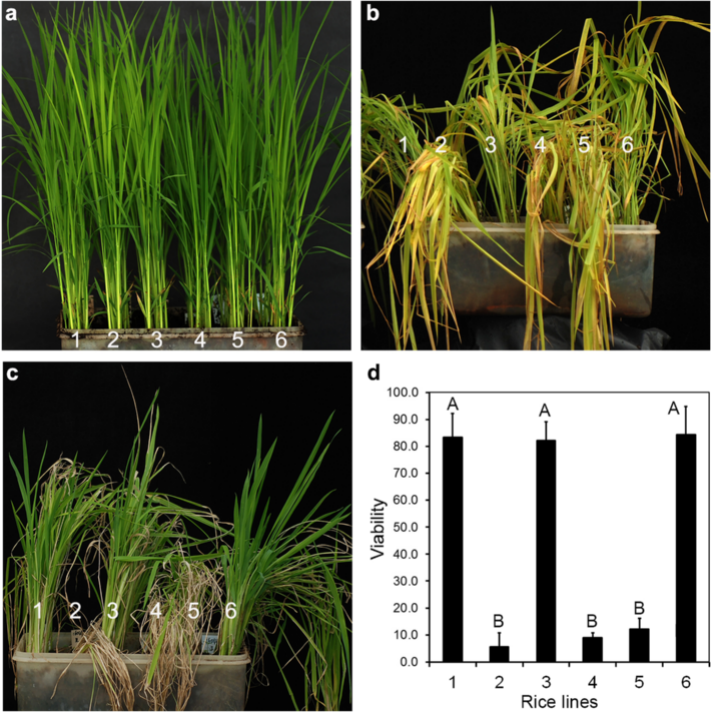
Evaluation of rice lines for submergence tolerance. **a** Phenotypes of 14-day-old plants before submergence treatment. **b** Phenotypes of plants after 14 days submergence treatment. **c** Phenotypes of plants after 7 days of recovery from 14 days of submergence treatment. **d** Viability of plants after 7 days of recovery from 14 days of submergence treatment. The data represents mean ± SD (*n* = 3, 30 plants in each treatment). The different letters (A or B) show significant difference among varieties at *P* = 0.01 levels of probability according to Duncan’s multiple range tests. 1, IR64(*Sub1ASub1A*); 2, MH725; 3, WH6725; 4, II-32A; 5, II-32A/MH725; 6, II-32A/WH6725

Two-week-old seedlings of WH6725, its hybrid rice and the control rice lines were tested for submergence tolerance (Fig. 4a). After 14 days of submergence, MH725, II-32A and II-32A/MH725 plants turned yellow, while IR64(*Sub1ASub1A*), WH6725 and II-32A/WH6725 plants remained in light green (Fig. 4b). The submergence-tolerant plants started to grow again when they were shifted to normal growth condition (Fig. 4c). Plant viability was scored based on the emergence and growth of green leaves over 7 days of recovery after 14 days of submergence (Fig. 4d). Most of the IR64 (*Sub1ASub1A*) (viability = 83.3 ± 8.8%), WH6725 (viability = 82.2 ± 6.9%) and II-32A/WH6725 (viability = 84.4 ± 10.2%) plants recovered and survived after 14 days of submergence, whereas most of the MH725 (viability = 5.6 ± 5.1%), II-32A (viability = 8.9 ± 1.9%) and II-32A/MH725 (viability = 12.2 ± 3.8%) plants died, with a few plants survived but in poor health (Fig. 4c and d). The results demonstrated that both WH6725 and II-32A/WH6725 provided submergence tolerance for over 14 days at 2-week-old seedling stage.

### Field trials and major important agronomic traits of WH6725 and its hybrid rice

To test the performance of rice lines in field condition, four field trials were conducted for WH6725 and MH725. Meanwhile, two field trials were conducted for II-32A/WH6725 and II-32A/MH725. The major important agronomic traits were collected from plants in field trials. WH6725 had similar growth duration to that of MH725 in the 4 field trials (Table 3). Similar length of growth duration was also recorded between II-32A/WH6725 and II-32A/MH725 in Field trial 3 and Field trial 4 (Table 3). The plant height of WH6725 was 0.6 to 8.9 cm higher than that of MH725 in the 4 field trials (Table 3). However, the plant height of II-32A/WH6725 was similar to that of II-32A/MH725 in Field trial 3 and Field trial 4 (Table 3). In all field trials, WH6725 and II-32A/WH6725 produced similar number of effective panicles per plant, similar panicle size and similar number of spikelets per panicle to MH725 and II-32A/MH725, respectively (Table 3). In addition, WH6725 and II-32A/WH6725 had similar spikelet fertility to MH725 and II-32A/MH725, respectively (Table 3). These results indicated that WH6725 can be used as the restorer line in 3-line hybrid rice production. Preferably, the 1000-grain weight of WH6725 was slightly heavier than that of MH725 in Field trials 2, 3 and 4 (*P*-value ranged from 0.00 to 0.05) (Table 3). The same phenomenon was observed in hybrid rice in Field trials 3 and 4 (*P*-value: 0.04, 0.07) (Table 3). Finally, the yield of WH6725 and II-32A/WH6725 were similar to that of MH725 and II-32A/MH725, respectively (Table 3). In general, the field trail results demonstrated that the major important agronomic traits of WH6725 and II-32A/WH6725 were similar to those of MH725 and II-32A/MH725, respectively.

**Table 3:** Major important agronomic traits of MH725, WH6725 and hybrid rice

Field trial^a^	Variety^b^	Growth duration (days)	Plant height (cm)	Number of effective panicles/plant	Panicle length (cm)	Total number of spikelets/panicle	Spikelet fertility (%)	1000-grain weight (g)	Yield/plant (g)
1	MH725 (I)	159	91.5 ± 1.4	8.6 ± 0.9	22.7 ± 0.4	167.3 ± 14.3	88.9 ± 1.1	29.7 ± 0.9	38.4 ± 8.2
	WH6725 (I)	160	92.1 ± 1.3	7.7 ± 0.9	22.0 ± 0.4	154.7 ± 5.1	90.9 ± 3.7	29.4 ± 0.3	31.9 ± 3.4
	*P*-value^c^	-	0.76	0.24	0.58	0.50	0.45	0.70	0.40
2	MH725 (I)	154	93.4 ± 0.3	5.1 ± 0.3	21.4 ± 0.7	157.3 ± 27.2	91.7 ± 1.4	27.7 ± 0.5	21.6 ± 2.0
	WH6725 (I)	155	98.5 ± 1.3*	5.0 ± 0.2	21.5 ± 0.1	149.7 ± 2.8	91.0 ± 1.2	29.8 ± 0.6*	20.5 ± 0.9
	*P*-value	-	0.03	0.42	0.75	0.70	0.71	0.05	0.20
3	MH725 (I)	138	121.2 ± 2.1	6.7 ± 1.0	26.8 ± 0.5	216.1 ± 11.2	72.1 ± 5.8	26.1 ± 0.3	27.3 ± 4.2
	WH6725 (I)	139	130.1 ± 2.6**	6.9 ± 0.9	26.9 ± 1.6	182.4 ± 44.5	77.6 ± 9.9	28.3 ± 0.4**	26.9 ± 2.6
	*P*-value	-	0.01	0.30	0.95	0.32	0.18	0.00	0.93
	II-32A/MH725 (H)	139	145.1 ± 6.3	6.4 ± 1.3	30.3 ± 3.0	226.3 ± 21.6	77.9 ± 4.1	26.4 ± 0.1	29.4 ± 3.7
	II-32A/WH6725 (H)	140	145.1 ± 4.7	6.3 ± 1.0	28.5 ± 0.8	245.9 ± 31.8	84.8 ± 5.5	27.0 ± 0.3	35.1 ± 3.5
	*P*-value	-	0.98	0.88	0.31	0.12	0.32	0.07	0.25
4	MH725 (I)	136	115.2 ± 0.3	7.4 ± 0.1	28.4 ± 0.6	217.8 ± 43.4	87.9 ± 1.3	26.5 ± 0.4	38.2 ± 7.4
	WH6725 (I)	136	122.6 ± 1.1**	7.5 ± 0.7	27.0 ± 0.3**	183.6 ± 7.6	88.6 ± 2.4	29.1 ± 0.4**	35.2 ± 2.3
	*P*-value	-	0.01	0.72	0.01	0.35	0.77	0.00	0.47
	II-32A/MH725 (H)	138	131.6 ± 1.1	7.4 ± 0.6	26.8 ± 1.4	299.5 ± 74.1	91.3 ± 2.7	26.9 ± 0.4	45.8 ± 3.0
	II-32A/WH6725 (H)	138	131.9 ± 0.8	6.7 ± 0.6	26.4 ± 0.2	220.1 ± 4.7	90.0 ± 3.8	27.9 ± 0.6*	38.2 ± 3.1
	*P*-value	-	0.69	0.09	0.61	0.22	0.61	0.04	0.12

*, **Stand for significance at 0.01 and 0.05 probability levels, respectively

^a^Field trial 1 was conducted in Lingshui, Hainan, China, in the winter season from November 2013 to April 2014. Plant spaced at 13 cm × 25 cm coupled a seedling per hill. Field trial 2 was conducted in Lingshui, Hainan, China, in the winter season from November 2014 to April 2015. Plants spaced at 13 cm × 17 cm coupled a seedling per hill. Field trial 3 was conducted in Hefei, Anhui, China, in the summer season from May 2014 to October 2014. Plant spaced at 16 cm × 17 cm coupled a seedling per hill. Field trial 4 was conducted in Hefei, Anhui, China, in the summer season from May 2015 to October 2015. Plant spaced at 13 cm × 27 cm coupled a seedling per hill

^b^I, inbred rice; H, hybrid rice

^c^ *P*-value for each trait was calculated in Microsoft Office Excel 2007 according to a two-tailed *t*-test for paired samples

### Grain quality of WH6725 and its hybrid rice

We evaluated the grain quality of WH6725, MH725, II-32A/WH6725 and II-32A/MH725 with the rice seeds harvested from the field trials. Both WH6725 and MH725 belong to the group of long grain rice (Grain length: WH6725, 7.0 ± 0.1 cm; MH725, 6.8 ± 0.1 cm) with medium grain shape (Ratio of length to width: WH6725, 3.0 ± 0.1; MH725, 2.9 ± 0.2) (Table 4). Both WH6725 and MH725 had the degree of chalkiness scored as 1 with slight variance in different field trials (Degree of chalkiness or DC: WH6725, 5.8 ± 5.4%; MH725, 4.7 ± 4.0%) (Table 4). They had low amylose content (Amylose content or AC: WH6725, 15.6 ± 1.2%; MH725, 16.3 ± 1.3%) and soft gel consistency (GC) (>60 mm) (GC: WH6725, 80.5 ± 7.6 mm; MH725, 76.2 ± 5.2 mm) (Table 4). Both WH6725 and MH725 had high alkali spreading values (ASV) (ASV: WH6725, 6.4 ± 0.5; MH725, 6.3 ± 0.3) and low gelatinization temperature (< 70 ^o^C) (Table 4). In addition, WH6725 had strong aromatic fragrance, whereas MH725 was tested to be a non-aromatic rice (Table 4). For hybrid rice, both II-32A/WH6725 and II-32A/MH725 belong to the group of medium grain rice (Grain length: II-32A/WH6725, 6.1 ± 0.3 cm; II-32A/MH725, 6.4 ± 0.2 cm) with medium grain shape (Ratio of length to width: II-32A/WH6725, 2.5 ± 0.0; 32A/MH725, 2.6 ± 0.2) (Table 4). Both II-32A/WH6725 and II-32A/MH725 had the degree of chalkiness scored as 5 (DC: II-32A/WH6725, 10.5 ± 3.0%; II-32A/MH725, 13.5 ± 8.0%) (Table 4). They also had intermediate amylose content (AC: II-32A/WH6725, 23.8 ± 0.1%; II-32A/MH725, 23.4 ± 0.4%) and soft gel consistency (> 60 mm) (GC: II-32A/WH6725, 60.0 ± 17.0 mm; II-32A/MH725, 69.5 ± 7.8 mm) (Table 4). In addition, both II-32A/WH6725 and II-32A/MH725 had high alkali spreading values (ASV: II-32A/WH6725, 6.2 ± 0.2; II-32A/MH725, 5.8 ± 1.1) and low gelatinization temperature (< 70 ^o^C) (Table 4). It should be noted that II-32A/WH6725 was characterised to be a non-aromatic rice due to the presence of the heterozygous *badh2.1* gene in the F1 hybrids. In the future, an aromatic hybrid rice could be produced by crossing WH6725 with an aromatic CMS or TGMS line. Nevertheless, the results collectively indicated that both WH6725 and II-32A/WH6725 retained similar grain quality to MH725 and II-32A/MH725, respectively, in terms of physical properties, cooking and eating quality.

**Table 4:** Grain quality of MH725, WH6725 and hybrid rice^a^

Trait	Inbred rice			Hybrid rice		
	MH725	WH6725	*P*-Value	II-32A/MH725	II32A/WH6725	*P*-Value
Grain length (mm)^b^	6.8 ± 0.1 (Long)	7.0 ± 0.1 (Long)	0.04	6.4 ± 0.2 (Medium)	6.1 ± 0.3 (Medium)	0.13
Ratio of length to width^c^	2.9 ± 0.2 (Medium)	3.0 ± 0.1 (Medium)	0.39	2.6 ± 0.2 (Medium)	2.5 ± 0.0 (Medium)	0.80
Degree of chalkiness (%)^d^	4.7 ± 4.0 (1)	5.8 ± 5.4 (1)	0.23	13.5 ± 8.0 (5)	10.5 ± 3.0 (5)	0.56
Amylose content (%)^e^	16.3 ± 1.3 (Low)	15.6 ± 1.2 (Low)	0.19	23.4 ± 0.4 (Intermediate)	23.8 ± 0.1 (Intermediate)	0.50
Gel consistency (mm)^f^	76.2 ± 5.2 (Soft)	80.5 ± 7.6 (Soft)	0.21	69.5 ± 7.8 (Soft)	60.0 ± 17.0 (Soft)	0.68
Alkali spreading value and Gelatinization temperature^g^	6.3 ± 0.3, GT <70 ^o^C (Low)	6.4 ± 0.5, GT < 70 ^o^C (Low)	0.50	5.8 ± 1.1, GT < 70 ^o^C (Low)	6.2 ± 0.2, GT < 70 ^o^C (Low)	0.63
Fragrance^h^	Non-aromatic	Strongly aromatic	-	Non-aromatic	Non-aromatic	

^a^The rice grain quality was evaluated for four times using the rice seeds harvested in 4 field trials as described in Table 2

^b^Category of grain length: Very long, grain length (GL) > 7.5 mm; Long, 6.6 mm < GL ≤ 7.5 mm; Medium, 5.5 mm < GL ≤ 6.6 mm; Short, GL ≤ 5.5 mm

^c^Ratio of length to width (L/W ratio): Slender, L/W ratio > 3.0; Medium, 2.0 < L/W ratio ≤ 3.0; Bold, L/W ratio, ≤ 2.0

^d^Scale for degree of chalkiness (DC): 0, DC = 0; 1, 0 < DC ≤ 10%; 5, 10% < DC ≤ 20%; 9, DC > 20%

^e^Classification of amylose content (AC): Waxy, AC ≤ 2%; Very low, 2% < AC ≤10%; Low, 10% < AC ≤ 20%; Intermediate, 20% < AC ≤ 25%; High, AC > 25%

^f^Classification of gel consistency (GC): Soft, GC > 60 mm; Medium, 40 mm < GC ≤ 60 mm; Hard, GC ≤ 40 mm

^g^Grade of gelatinization temperature (GT) estimated by alkali spreading value (ASV): High, 74.5 °C ≤ GT < 80 °C, 1 ≤ ASV < 2.5; Intermediate high, 74 °C ≤ GT < 74.5 °C, 2.5 ≤ ASV < 3.5; Intermediate, 70 °C ≤ GT < 74 °C, 3.5 ≤ ASV < 5.5; Low, GT < 70 °C, 5.5 ≤ ASV ≤ 7

^h^Scale of fragrance was characterised as strongly aromatic, moderately aromatic, slightly aromatic and non-aromatic as described previously (Cruz and Khush 2000)

## Discussion

MAS technology has greatly facilitated and accelerated the breeding of WH6725 in this study. Firstly, MAS is a very efficient and cost-effective technology for breeding as it is used in selection for most of the steps of breeding program. As a result, disease evaluation and submergence tolerance test are only being performed at the final step of the breeding study, thereby reducing time and cost. Secondly, MAS enabled us to select the *badh2.1* gene in backcrossing progeny as the *badh2.1* gene controls the aromatic trait in recessive inheritance and the phenotypic selection cannot be done in heterozygous plants. Thirdly, MAS allowed us to pyramid the *Xa4*, *Xa21* and *Xa27* genes in a single rice line WH6725 without disease evaluation using *R* gene-specific *X. oryzae* pv. *oryzae* strains. Although the 3 dominant *R* genes are all for disease resistance to rice bacterial blight, each of them has its unique resistance specificity and their resistance spectrums are highly overlapped (Gu et al. 2004; Luo et al. 2012). With the help of MAS technology, we first combined the *Xa4* and *Xa21* genes in WH421 in the previous report (Luo et al. 2012) and then pyramided the *Xa4*, *Xa21* and *Xa27* genes in WH6725 in this study. Finally, MAS technology provided precise selection and accelerated the breeding process. With the molecular markers developed from the selected genes or closely linked to them, we could precisely identify the 4 F2 plants that contained homozygous alleles at the *badh2.1*, *Pi9*, *Sub1A*, *Xa4*, *Xa21* and *Xa27* loci from a manageable population consisting of 960 individuals (Fig. 1). The selection of all genes was done in one generation, which could not be achieved through phenotypic selection. Phenotypic evaluation of WH6725 and F1 hybrids for disease resistance, submergence tolerance and fragrance confirmed the genotype of WH6725 determined by the molecular markers. Starting from WH421 and through MAS, we spent four years or 10 generations on breeding WH6725. Compared to conventional breeding based on phenotypic selection, the MAS technology saved us at least 2 years in the breeding of WH6725.

Single *R* gene for disease resistance might be easily broken down by the emergence of new races or strains of pathogens (Cruz et al. 2000). The probability of pathogen to overcome two or more effective genes by mutation is much lower compared to the ‘attacking’ of resistance controlled by a single gene. Previous report demonstrated that the combination of the *xa5*, *xa13* and *Xa21* genes in indica rice cultivar PR106 could provide broad spectrum resistance to different *X. oryzae* pv. *oryzae* races or isolates (Singh et al. 2001). Three bacterial blight *R* genes, *Xa4*, *Xa21* and *Xa27*, were pyramided in WH6725 and bacterial blight inoculation demonstrated that WH6725 and its hybrid rice could provide high and broad spectrum resistance to multiple *X. oryzae* pv. *oryzae* strains. Among the three *R* genes, the *Xa4* gene provides durable disease resistance at all developmental stages to bacterial blight pathogens and has been used in Asian rice breeding for many years (Zhang 2009). Both *Xa21* and *Xa27* confer broad-spectrum disease resistance to *X. oryzae* pv. *oryzae* strains and their molecular mechanisms for disease resistance are different (Gu et al. 2004; Gu et al. 2005; Ikeda et al. 1990; Song et al. 1995). Moreover, the pyramiding of the *Pi9*, *Xa4*, *Xa21* and *Xa27* genes in WH6725 will not only provide disease resistance to rice blast and bacterial blight but also reduce the usage of bactericides and fungicides. Together with the *Sub1A* gene for submergence tolerance, WH6725 and its hybrid rice could be regarded as the environment-friendly rice with adaptability to unfavourable climate change due to global warming.

It is important that the genes to be employed in marker-assisted breeding and gene pyramiding should not bring in undesirable traits due to linkage drag (Peng et al. 2014a, b; Sun and Mumm 2015; Wang et al. 2015). In addition to fragrance, the major important agronomic traits of WH6725 were similar to that of the initial recurrent female line MH725. The results indicate that no deleterious effect presents in WH6725 after the introgression and pyramiding of the six genes. Previous reports also revealed that the pyramiding of disease resistance genes and submergence tolerance gene in rice did not compromise the yield or grain quality (Chen et al. 2000; Luo and Yin 2013; Neeraja et al. 2007). High yield, multi-resistance or tolerance to biotic and abiotic stresses and good grain quality are ultimate goals for rice breeding (Zhang 2007). The breeding of WH6725 with disease resistance to rice blast and bacterial blight and submergence tolerance would contribute to the stability and sustainability of hybrid rice production.

## Conclusion

An introgression rice line mainly in MH725 genetic background, designed as WH6725, has been developed to contain bacterial blight resistance genes *Xa4*, *Xa21* and *Xa27*, blast resistance gene *Pi9*, submergence tolerance gene *Sub1A* and fragrance gene *badh2.1* through MAS and gene pyramiding. The development of WH6725 provides an improved restorer line for hybrid rice production with disease resistances to rice blast and bacterial blight, tolerance to submergence and good grain quality with aromatic fragrance.

## Methods

### Rice cultivars

MH725 is an elite restorer line that has been widely used to produce three-line hybrid rice in China. WH421 is an introgression rice line that carries *Xa4* and *Xa21* gene in MH725 genetic background (Luo et al. 2012) and was used as the recurrent female line in this study. KDML105 is the most popular cultivar of aromatic rice grown in Thailand and was used as the donor line for the *badh2.1* gene (Bradbury et al. 2005; Kovach et al. 2009; Luo and Yin 2013). IRBB27 is a near-isogenic line (NIL) of *Xa27* in IR24 genetic background (Gu et al. 2004). 75-1-127 is an introgression rice line that carries rice blast *R* gene *Pi9* in IR31917 genetic background (Liu et al. 2002). IR64 (*Sub1ASub1A*) is an introgression rice line that carries *Sub1A* in IR64 genetic background (Luo and Yin 2013). II-32A is a CMS line, which is broadly used as the maternal line to produce three-line hybrid rice. 1892S is a TGMS rice line, which is broadly used as the maternal line to produce two-line hybrid rice.

### PCR-based molecular markers and PCR conditions

The molecular markers used for MAS in this study are listed in Table 1. The molecular markers for the *badh2.1* and *Badh2* alleles were the dominant and allelic-specific makers M265 and M355, respectively (Table 1). The molecular marker for the *Pi9* gene was the co-dominant sequence-tagged-site (STS) marker NBS2-1, which was derived from the *Pi9* gene (Table 1). The molecular marker for the *Sub1A* gene was the co-dominant simple sequence repeat (SSR) marker RM23887 (Neeraja et al. 2007). The molecular marker for the *Xa4* gene was the co-dominant SSR marker RM224, which is 1.1 cM from the *Xa4* locus (Sun et al. 2003). The molecular marker for the *Xa21* gene was the co-dominant STS marker 21, which was derived from the *Xa21* gene (Chen et al. 2000). The molecular marker for the *Xa27* gene was the co-dominant STS marker M124, which is located at 43 kb upstream to the *Xa27* gene.

PCR amplification of molecular markers was performed on a PTC-100 programmable thermal controller (MJ Research, USA). The PCR reaction mixture of 20 μl consisted of 10 ng of rice genomic DNA, 0.15 mM of each dNTP, 0.15 mM of each primer, 2 μl of 10 × PCR buffer and 1 unit of Taq polymerase. Template DNA was initially denatured at 94 °C for 2 mins followed by 35 cycles of PCR amplification with the following parameters: 30 s of denaturation at 94 °C, 40 s of primer annealing at 52 °C for RM23887, 55 °C for 21 and RM224, 58 °C for M265 and M355, 60 °C for M124 and NBS2-1, and 1 min of primer extension at 72 °C. Finally, the reaction mixture was maintained at 72 °C for 5 mins before completion. M265 and M355 were amplified separately and mixed for each sample loading on agarose gel. The PCR products were electrophoretically resolved on a 1.5% agarose gel for NBS2-1, a 2.0% agarose gel for 21, M265 and M355, and a 3.5% agarose gel for markers RM23887, RM224 and M124 in 1 × TAE buffer.

### Rice blast inoculation

Isolates of *M. oryzae,* ZB13, 11-3-1-1-2, 11-17-1-2 and M39-1-3-8-1, were used for inoculation experiments. The first three *M. oryzae* isolates were collected from Anhui, China. M39-1-3-8-1 was collected from the Philippines. *M. oryzae* isolates were grown on prune agar medium (40 ml prune juice, 20 g/l agar, 5 g/l sucrose, 5 g/l lactose, 1 g/l yeast extract, pH6.5) at 28 °C in darkness for 7 days and at 26 °C under light for 3–5 days. The rice plants at 4-leaf-stage were inoculated with a spore suspension at the density of 1 × 10^5^ spores/ml. The inoculated plants were immediately placed in a dew chamber for 24 h at 25 °C and 90% of humidity in darkness. The plants were then transferred to a growth chamber at 25–28 °C under a 12/12 h (light/dark) photoperiod and 90% humidity. Disease phenotypes were photographed at 7 days after inoculation.

### Bacterial blight inoculation

*X. oryzae* pv*. oryzae* strains were grown on PSA medium (10 g/l peptone, 10 g/l sucrose, 1 g/l glutamic acid, 16 g/l bacto-agar, and pH7.0) at 28 °C for 2 days. The bacterial cells were collected and suspended in sterile water at an optical density of 0.5 (OD600). Bacterial blight inoculation was carried out according to the leaf-clipping method (Kauffman et al. 1973). Disease scoring was measured as described previously (Gu et al. 2004).

### Test of rice for submergence tolerance

Test of rice plants for submergence tolerance was conducted in open water tanks in greenhouse. About 30 2-week-old plants of each line were completely submerged in water for 14 days. After treatment, the plants were transferred to greenhouse for recovery for 7 days and then were scored for viability. The plant survival is indicated by having at least one green leaf. The statistical analysis was performed by Duncan’s multiple range tests (Duncan 1955). The experiments were repeated for three times.

### Design of field trials and measurement of major important agronomic traits

Four field trials were implemented with MH725, WH6725 and their hybrid rice. Field trial 1 and Field trial 2 were conducted in Lingshui (Hainan, China) in the winter seasons (November–April) of 2013/2014 and 2014/2015, respectively. Field trial 3 and Field trial 4 were carried out in Hefei (Anhui, China) in the summer seasons (May–October) of 2014 and 2015, respectively. In each field trial, paired parental lines and hybrid rice were arranged near to each other in the plots at the size of at least 12 m^2^. Three repeat plots were planted for each parental line or hybrid rice line.

The major important agronomic traits of rice, including growth duration, plant height, effective panicle number per plant, panicle length, total spikelet number per panicle, spikelet fertility, 1000-grain weight and yield per plant, were obtained from 10 plants grown in each plot and a total of 30 plants in 3 plots were measured for each variety. The growth duration was counted based on the number of days from sowing to 85% maturity per panicle in 90% of the plants population in a plot. The plant height was measured from soil surface to tip of the tallest panicle (awns excluded) at one day before harvest or at harvest. The number of effective panicle number per plant was the total number of panicles in a plant that produced more than 5 grains. The panicle length was measured from the base to the tip of a panicle (awns excluded). The total spikelet number per panicle contained the number of both filled and empty grains in a panicle. The spikelet fertility was the percentage of the number of the filled grains over the number of the total spikelet in a plant. The 1000-grain weight was measured with 1000 sun-dried filled grains. The yield per plant was the average weight of the filled grains per plants. P-value for each trait was calculated in Microsoft Office Excel 2007 according to a two-tailed *t*-test for paired samples.

### Evaluation of grain quality

The grain quality was evaluated with the rice grains harvested from the four field trials mentioned above. Rice grain quality properties, including grain length, ratio of length/width, degree of chalkiness, amylose content, gel consistency, alkali spreading value and fragrance, were measured according to the methods described previously (Cruz and Khush 2000). P-value for each characteristic was calculated in Microsoft Office Excel 2007 according to a two-tailed *t*-test for paired samples.

## Abbreviations

*BADH2*: Betaine aldehyde dehydrogenase gene
CMS: Cytoplasmic male sterile
MAS: Marker-assisted selection
MH725: Mianhui 725
PCR: Polymerase chain reaction
*R*: Resistance gene
STS: Sequence-tagged site
TGMS: Thermosensitive-genic male sterility
WH421: Wanhui 421
WH6725: Wanhui 6725.

## References

[CR1] Amante-Bordeos A, Sitch LA, Nelson R, Dalmacio RD, Oliva NP, Aswidinnoor H, Leung H (1992). Transfer of bacterial blight and blast resistance from the tetraploid wild rice Oryza minuta to cultivated rice, Oryza sativa. Theor Appl Genet.

[CR2] Blair MW, McCouch SR (1997). Microsatellite and sequence-tagged site markers diagnostic for the rice bacterial leaf blight resistance gene *xa-5*. Theor Appl Genet.

[CR3] Bradbury LM, Fitzgerald TL, Henry RJ, Jin Q, Waters DL (2005). The gene for fragrance in rice. Plant Biotechnol J.

[CR4] Chen S, Lin X, Xu C, Zhang Q (2000). Improvement of bacterial blight resistance of ‘Minghui 63’, an elite restorer line of hybrid rice, by molecular marker-assisted selection. Crop Sci.

[CR5] Cruz ND, Khush GS, Singh RK, Singh US, Khush GS (2000). Rice grain quality evaluation procedures. Aromatic rices.

[CR6] Cruz CMV, Bai J, Oña I, Leung H, Nelson RJ, Mew T-W, Leach JE (2000). Predicting durability of a disease resistance gene based on an assessment of the fitness loss and epidemiological consequences of avirulence gene mutation. Proc Natl Acad Sci U S A.

[CR7] Datta K, Baisakh N, Thet KM, Tu J, Datta SK (2002). Pyramiding transgenes for multiple resistance in rice against bacterial blight, yellow stem borer and sheath blight. Theor Appl Genet.

[CR8] Duncan DB (1955). Multiple range and multiple F tests. Biometrics.

[CR9] Fukao T, Yeung E, Bailey-Serres J (2011). The submergence tolerance regulator SUB1A mediates crosstalk between submergence and drought tolerance in rice. Plant Cell.

[CR10] Gnanamanickam S, Priyadarisini VB, Narayanan N, Vasudevan P, Kavitha S (1999). An overview of bacterial blight disease of rice and strategies for its management. Curr Sci.

[CR11] Gu K, Tian D, Yang F, Wu L, Sreekala C, Wang D, Wang GL, Yin Z (2004). High-resolution genetic mapping of *Xa27 (t)*, a new bacterial blight resistance gene in rice, *Oryza sativa L*. Theor Appl Genet.

[CR12] Gu K, Yang B, Tian D, Wu L, Wang D, Sreekala C, Yang F, Chu Z, Wang G-L, White FF (2005). R gene expression induced by a type-III effector triggers disease resistance in rice. Nature.

[CR13] Huang N, Angeles ER, Domingo J, Magpantay G, Singh S, Zhang G, Kumaravadivel N, Bennett J, Khush GS (1997). Pyramiding of bacterial blight resistance genes in rice: marker-assisted selection using RFLP and PCR. Theor Appl Genet.

[CR14] Ikeda R, Khush G, Tabien R (1990). A new resistance gene to bacterial blight derived from *O. longistaminata*. Jpn J Breed.

[CR15] Kauffman H, Reddy A, Hsieh S, Merca S (1973). An improved technique for evaluating resistance of rice varieties to *Xanthomonas oryzae*. Plant Dis Rep.

[CR16] Kaundal R, Kapoor AS, Raghava GP (2006). Machine learning techniques in disease forecasting: a case study on rice blast prediction. BMC Bioinformatics.

[CR17] Khanna A, Sharma V, Ellur RK, Shikari AB, Gopala Krishnan S, Singh UD, Prakash G, Sharma TR, Rathour R, Variar M, Prashanthi SK, Nagarajan M, Vinod KK, Bhowmick PK, Singh NK, Prabhu KV, Singh BD, Singh AK (2015). Development and evaluation of near-isogenic lines for major blast resistance gene(s) in Basmati rice. Theor Appl Genet.

[CR18] Koide Y, Ebron LA, Kato H, Tsunematsu H, Telebanco-Yanoria MJ, Kobayashi N, Yokoo M, Maruyama S, Imbe T, Fukuta Y (2011). A set of near-isogenic lines for blast resistance genes with an Indica-type rainfed lowland elite rice (*Oryza sativa L.*) genetic background. Field Crop Res.

[CR19] Kottapalli KR, Narasu ML, Jena KK (2010). Effective strategy for pyramiding three bacterial blight resistance genes into fine grain rice cultivar, Samba Mahsuri, using sequence tagged site markers. Biotechnol Lett.

[CR20] Kovach MJ, Calingacion MN, Fitzgerald MA, McCouch SR (2009). The origin and evolution of fragrance in rice (Oryza sativa L.). Proc Natl Acad Sci U S A.

[CR21] Liu G, Lu G, Zeng L, Wang GL (2002). Two broad-spectrum blast resistance genes, *Pi9(t)* and *Pi2(t)*, are physically linked on rice chromosome 6. Mol Genet Genomics.

[CR22] Liu W, Liu J, Triplett L, Leach JE, Wang G-L (2014). Novel insights into rice innate immunity against bacterial and fungal pathogens. Annu Rev Phytopathol.

[CR23] Luo Y, Yin Z (2013) Marker-assisted breeding of Thai fragrance rice for semi-dwarf phenotype, submergence tolerance and disease resistance to rice blast and bacterial blight. Mol Breed 32:709-721

[CR24] Luo Y, Sangha J, Wang S, Li Z, Yang J, Yin Z (2012). Marker-assisted breeding of *Xa4*, *Xa21* and *Xa27* in the restorer lines of hybrid rice for broad-spectrum and enhanced disease resistance to bacterial blight. Mol Breed.

[CR25] Luo Y, Zakaria S, Basyah B, Ma T, Li Z, Yang J, Yin Z (2014). Marker-assisted breeding of Indonesia local rice variety Siputeh for semi-dwarf phonetype, good grain quality and disease resistance to bacterial blight. Rice (N Y).

[CR26] Mew T, Alvarez A, Leach J, Swings J (1993). Focus on bacterial blight of rice. Plant Dis Rep.

[CR27] Neeraja CN, Maghirang-Rodriguez R, Pamplona A, Heuer S, Collard BC, Septiningsih EM, Vergara G, Sanchez D, Xu K, Ismail AM, Mackill DJ (2007) A marker-assisted backcross approach for developing submergence-tolerant rice cultivars. Theor Appl Genet 115:767–776 10.1007/s00122-007-0607-0 17657470

[CR28] Ni D, Song F, Ni J, Zhang A, Wang C, Zhao K, Yang Y, Wei P, Yang J, Li L (2015). Marker-assisted selection of two-line hybrid rice for disease resistance to rice blast and bacterial blight. Field Crop Res.

[CR29] Peng T, Sun X, Mumm RH (2014). Optimized breeding strategies for multiple trait integration: I. Minimizing linkage drag in single event introgression. Mol Breed.

[CR30] Peng T, Sun X, Mumm RH (2014). Optimized breeding strategies for multiple trait integration: II. Process efficiency in event pyramiding and trait fixation. Mol Breed.

[CR31] Perez LM, Redona ED, Mendioro MS, Cruz CMV, Leung H (2008). Introgression of *Xa4*, *Xa7* and *Xa21* for resistance to bacterial blight in thermosensitive genetic male sterile rice (Oryza sativa L.) for the development of two-line hybrids. Euphytica.

[CR32] Rajpurohit D, Kumar R, Kumar M, Paul P, Awasthi A, Basha PO, Puri A, Jhang T, Singh K, Dhaliwal HS (2011). Pyramiding of two bacterial blight resistance and a semidwarfing gene in Type 3 Basmati using marker-assisted selection. Euphytica.

[CR33] Sarkarung S, Somrith B, Chitrakorn S, Singh R, Singh U, Khush G (2000). Aromatic rice of Thailand. Aromatic rices.

[CR34] Singh S, Sidhu JS, Huang N, Vikal Y, Li Z, Brar DS, Dhaliwal HS, Khush GS (2001). Pyramiding three bacterial blight resistance genes (*xa5*, *xa13* and *Xa21*) using marker-assisted selection into indica rice cultivar PR106. Theor Appl Genet.

[CR35] Somrith B (1996). Khao Dawk Mali 105: Problems, research efforts and future prospects. Report of the INGER Monitoring Visit on Fine-Grain Aromatic Rice in India, Iran, Pakistan and Thailand.

[CR36] Song W-Y, Wang G-L, Chen L-L, Kim H-S (1995). A receptor kinase-like protein encoded by the rice disease resistance gene, Xa21. Science.

[CR37] Spielmeyer W, Ellis MH, Chandler PM (2002). Semidwarf (*sd-1*), “green revolution” rice, contains a defective gibberellin 20-oxidase gene. Proc Natl Acad Sci U S A.

[CR38] Sun X, Mumm RH (2015). Optimized breeding strategies for multiple trait integration: III. Parameters for success in version testing. Mol Breed.

[CR39] Sun X, Yang Z, Wang S, Zhang Q (2003). Identification of a 47-kb DNA fragment containing Xa4, a locus for bacterial blight resistance in rice. Theor Appl Genet.

[CR40] Wang Q, He G (2007). Genetic divergence between rice germplasms of blast resistance and the major WA-CMS-type restorers. Mol Plant Breed.

[CR41] Wang X, Jia MH, Ghai P, Lee FN, Jia Y (2015). Genome-wide association of rice blast disease resistance and yield-related components of rice. Mol Plant Microbe Interact.

[CR42] Xu KN, Mackill DJ (1996). A major locus for submergence tolerance mapped on rice chromosome 9. Mol Breed.

[CR43] Xu K, Xu X, Fukao T, Canlas P, Maghirang-Rodriguez R, Heuer S, Ismail AM, Bailey-Serres J, Ronald PC, Mackill DJ (2006). *Sub1A* is an ethylene-response-factor-like gene that confers submergence tolerance to rice. Nature.

[CR44] Yuan L, Yang Z, Yang J, Virmani S (1994). Hybrid rice in China. Hybrid Rice Technology: New Developments and Future Prospects.

[CR45] Zhai WX, Wang WM, Zhou YL, Li XB, Zheng XW, Zhang Q, Wang GL, Zhu LH (2002). Breeding bacterial blight-resistant hybrid rice with the cloned bacterial blight resistance gene *Xa21*. Mol Breed.

[CR46] Zhang Q (2007). Strategies for developing Green Super Rice. Proc Natl Acad Sci U S A.

[CR47] Zhang Q (2009). Genetics and improvement of bacterial blight resistance of hybrid rice in China. Rice Sci.

[CR48] Zhang J, Li X, Jiang G, Xu Y, He Y (2006). Pyramiding of *Xa7* and *Xa21* for the improvement of disease resistance to bacterial blight in hybrid rice. Plant Breed.

